# Yaws Eradication: Facing Old Problems, Raising New Hopes

**DOI:** 10.1371/journal.pntd.0001837

**Published:** 2012-11-29

**Authors:** Andrea Rinaldi

**Affiliations:** Science Writer, Cagliari, Italy; University of Tennessee, United States of America

## An Old Acquaintance, a Troubled Relationship

The history of disease eradication coincides to a great deal with that of yaws. Soon after the World Health Organization (WHO) was established in 1948, yaws was the first disease to be slated for global eradication in the postwar era. Banking on a previous attempt to eliminate yaws in Haiti, where it was ravaging at that time—an initiative strongly sponsored by Fred Lowe Soper, the powerful director of the Pan American Health Organization (PAHO)—the Global Yaws Control Programme ran from 1952 to 1964, treating some 300 million people in 46 countries and reducing the global levels of the disease by 95%. “Different sources give different figures,” recently wrote medical historian Nancy Leys Stepan on this point [1]. “The variableness of the data in different sources indicates the inadequate reporting and surveillance mechanisms in place in many poor countries.” Indeed, after the initial success of the mass campaign, efforts lagged, failing to trace down and treat the last remaining cases. Also, subclinical infections were most likely overlooked, and contacts not adequately contained. As a consequence, yaws began to rapidly reappear in many regions.

**Figure 1:**
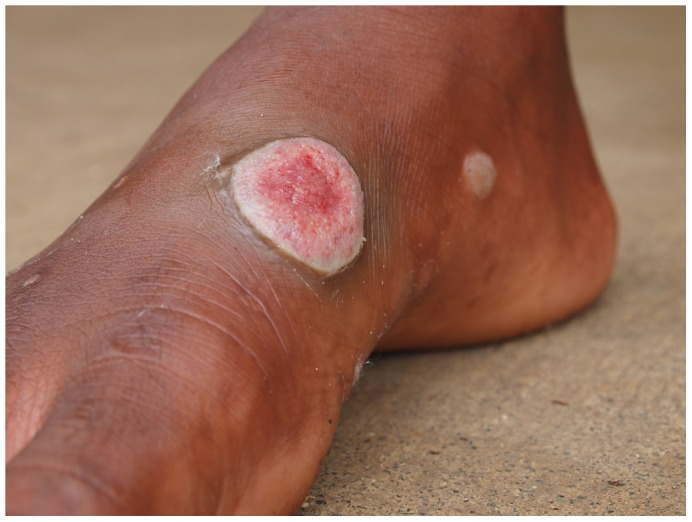
Typical yaws primary lesion, usually occurring in the limbs. Credit: Oriol Mitjà.

Yaws is a bacterial infection of the skin and bones (Figure 1), caused by the spirochete *Treponema pallidum* subspecies *pertenue* and transmitted by contact [2]. The yaws treponemes are very closely related (differ in less than 0.2% of the genome sequence) to syphilis treponemes (*T. pallidum* subspecies *pallidum*), although they are less virulent [3]. Yaws mainly affects children below 15 years of age living in poor rural settings in warm, tropical areas and, if not treated, can lead to gross disfigurement and disability in about 10% of cases. A single injection of long-acting penicillin—the silver bullet that became available after World War II and spurred the eradication projects mentioned above—can fully cure the disease. Distribution of the yaws is focalized, and well-validated, feasible, and widely available serological tests exist. Although these cannot differentiate between yaws and other *Treponema* infections such as syphilis, treponemal tests can be useful for screening in populations of low disease prevalence and as confirmatory tests to verify clinical observations. No vaccine exists, and no long-term immunization can be acquired upon treatment, and re-infection is thus possible. Humans are generally considered the sole reservoir, although a few reports exist of the presence of the disease among nonhuman primates, such as gorillas and baboons [4].

Official notification of yaws cases to WHO stopped in the 1990s, when control programmes where discontinued in most countries. At that time, the estimated global prevalence was at 2.5 million, with 460,000 new cases annually [5]. Nowadays, a few endemic countries in South-East Asia (Indonesia, Timor-Leste), the Pacific region (Papua New Guinea, Solomon Islands, Vanuatu), and Africa (Benin, Cameroon, Central African Republic, Côte d'Ivoire, Democratic Republic of the Congo, Ghana, Republic of the Congo, Togo) are believed to harbour the vast majority of cases [6], but figures are not clear due to patchy surveying, especially in isolated districts and islands, and “may only reflect the tip of the iceberg,” as WHO concedes [7]. Papua New Guinea reported some 28,989 cases in 2011, the Solomon Islands 20,635 in 2010, and Ghana 20,525 in 2010, while no recent accounts are available for Timor-Leste [6]. Besides, the current situation in many of the countries from where the disease was eliminated and that interrupted surveillance since then is virtually unknown.

**Figure 2:**
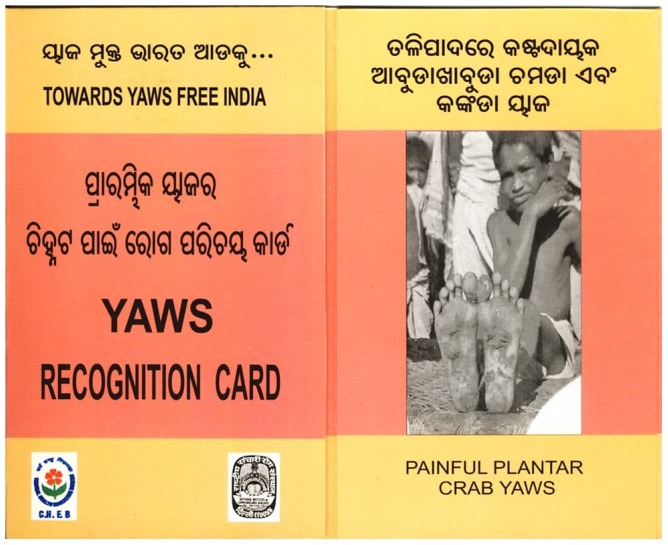
Yaws elimination in India. Scorecards, posters, and other illustrated material were largely distributed among both health workers and population to help spot remaining cases. Cash rewards were given to those residents who had reported suspected cases that were later serologically confirmed.

“If you can't eradicate yaws, which other disease can you possibly put down? Malaria? Forget about it!” This must be a recurrent thought for many of those dealing with neglected tropical diseases, and certainly has hovered in the mind of WHO officials for quite a long time. In the search for a confirmation (after smallpox) of the feasibility of the eradication approach, to be eventually “sold” to donors and institutions in support for by far more difficult-to-cope-with objectives, WHO never really gave up its war against yaws. In 2007, experts and delegates from endemic countries agreed on a renovated effort to assess yaws burden and restart activities to control the disease, eventually reaching elimination of clinical cases in South-East Asia by 2012 [8]. This revival came on the wake of a major achievement in India, where a very aggressive campaign (Figure 2) started in 1996, backed by strong political commitment, and permitted to declare yaws eliminated on September 2006, as no case of the disease was reported since 2003 [9]. The India campaign employed the strategy of selective community treatment with injected penicillin in an at-risk population of 7 million, and its success is clearly ascribable to an excellent and tenacious system for clinical and serological surveillance during and after completion of the program.

## Drafting a New Strategy (Being Aware of the Past)

Since then, however, not much has changed on the global scale. But a recent breakthrough could help in finding a way out in the ice cap that trapped the yaws eradication programme, boosting it forward. Last January, a study—an open-label, noninferiority, randomised trial conducted at Lihir island in Papua New Guinea and involving 250 children aged 6 months to 15 years with yaws—has shown that the patients in the group treated with a single oral dose (30 mg/kg) of azithromycin were cured as well as those receiving an intramuscular injection of 50,000 units/kg benzathine benzylpenicillin (96% versus 93%), as determined by both clinical and serological response [10]. “With yaws re-emerging, treatment with an effective drug that can be easily administered on a large scale is the preferred method for treatment, prevention, and, eventually, elimination worldwide,” wrote the authors [10]. “This is perhaps the most important publication on yaws in the past 50 years, and could facilitate the elimination of this ancient scourge,” remarked David Mabey from the London School of Hygiene & Tropical Medicine in an accompanying commentary [11]. A similar trial is in progress in Ghana, whose results are due in the near future. “The outcome of the serology tests is blinded and conclusions cannot be made as yet, but from our preliminary observations of the clinical outcome we are confident that the results will be in line with those obtained in Papua New Guinea,” said Cynthia Kwakye, municipal director of health services for Ga West Municipality, Ghana, and lead investigator.

Substituting a painful injection of penicillin with a single dose of an oral antibiotic could really be a significant advantage, as no trained staff would be needed to treat cases in remote areas, infection and anaphylactic shock control measures and other logistical problems linked to penicillin use would be overcome, and acceptability of treatment by those who receive it, especially children, would certainly improve. On the political side, switching to the new eradication strategy should similarly stimulate commitment and willingness to cooperate, signalling national governments that this will be not just a new attempt to complete the unfinished job using the same means (thus presumably ending the same way), but a motivatedly renovated effort to rid the world of a nasty disease, holding a new weapon (and a mighty one) in one hand and a better understanding of the necessary conditions in the other.

In the new treatment scheme, azithromycin in a single oral dose would be deployed at either clinically and/or serologically confirmed endemic village or community level, leaving benzathine penicillin as an alternative treatment [6]. Around the azithromycin pillar, therefore, the WHO now plans to wrap its renovated effort to eradicate yaws, a last call to arms that in the intentions of the organization should permit to reach zero cases in 2017, and the subsequent certification of worldwide interruption of transmission by 2020 [12]. A new eradication policy was sketched at a WHO consultation held in Morges, Switzerlnad, last March [6]. Besides moving from penicillin to azithromycin as a therapeutic tool, WHO is aware, other problems must be tackled, namely those that impeded completion of the eradication campaign back in the 1960s. So, where the 1950–60s campaign failed in identifying contacts of those infected and leaving subclinical infections to spread the disease again, the new strategy will enforce total mass treatment. “We are not going to treat an entire country, say, Ghana, but rather, if it is known that yaws is present in a community or village, total community treatment using oral azithromycin will be deployed there,” said Kingsley Asiedu, from the WHO's Department of Control of Neglected Tropical Diseases in Geneva. Also, to make sure all cases are tracked down and treated, WHO will require strict follow-up measures, with re-surveys conducted every 3–6 months and either total or selective community treatments as required plus active case finding, until zero case prevalence is reached. “It is critical that mass treatment campaigns for yaws are supported by ongoing surveillance, active case finding and treatment, including follow-up of close patient contacts,” said Lasse Vestergaard from WHO's Regional Office for the Western Pacific, Manila. “Although case numbers initially drop, they may soon come back up if the affected area is left unattended. We have seen in some places that the number of yaws cases reported from health facilities increased only within a few years after the implementation of a comprehensive mass treatment campaign, which achieved more than 90% coverage, but without any follow-up activities.” Post-zero case surveillance measures will include immediate investigation of all reported or rumoured cases, and yearly serological surveys in children aged 1–5 years, for 3 years [6]. Needless to say, with possible disease resurgence squatted in the dark [13], this last mile will be the most hard to run, and stopping transmission will require diligence, discipline, and total attention to details from all of those involved in field work and supervisors.

Clearly, getting a fresh idea of the epidemiological situation, especially in endemic areas with limited information (e.g., no Timor-Leste delegates were present at the Morges meeting), will be a must before getting involved in any ambitious eradication programme. Dealing with the integration of yaws activities into the existing general health services, at least where these exist, and a tireless work to promote community information and mobilization and health workers training, will also be key issues. “It is important that the entire primary health care system becomes involved and adequately strengthened to deal with yaws cases alongside so many other health problems. We need to ensure a balanced vertical and horizontal approach towards improving health,” said Vestergaard. “On the matter of vertical versus integrated programmes in public health, nearly every eradication campaign before the 1980s was seen as offering an entering ‘wedge’ for the development of more integrated or basic, everyday health care, but as it turned out, few of them did so,” said Stepan when questioned on this issue. “The relationship seems to be the other way around: the pre-existence of some kind of basic health care services, and a network of health officers, is what allowed a single-disease elimination and/or eradication drive to work. At any rate, today we seem to be moving towards a more integrated version of the vertical campaigns, and these can be effective.”

In order to overcome political apathy, moving rapidly ahead and getting momentum, the new campaign will need, the sooner the better, to receive the World Health Assembly's endorsement (the last WHA resolution, WHA 31.58, on yaws and other endemic treponematoses was released in 1978). A way to secure this would be to have a “proof-of-principle” that mass treatment using azithromycin is both doable and effective, and to this end standard operating guidelines will be developed by WHO and endemic countries will be asked to coordinate pilot studies to assess the impact of mass treatment of yaws using azithromycin in limited geographical areas. “The new strategy should be validated first in pilot studies, so that we can learn what is the impact in both clinical and subclinical infections in the treated population,” said Oriol Mitjà, the lead researcher behind the azithromycin trial in Papua New Guinea. Such pilot projects are scheduled to start in selected districts in Cameroon, Ghana, Indonesia, Papua New Guinea, the Solomon Islands, and Vanuatu, to move then the renewed eradication effort to other areas and countries within 2 years [6]. “If successful, it will demonstrate an effective, logistically feasible, safe, and acceptable protocol for global eradication of this neglected disease and we will be able to monitor potential macrolide resistance in yaws to ensure sustainability of the strategy,” said Mitjà. Indeed, a rapid spread of macrolide resistant *T. pallidum* strains has been recorded lately in places such as California, London, and Shanghai (e.g., [14]), so the massive use of azithromycin in the treatment of large populations raises concerns. However, it may be that such macrolide resistant bacterial strains “are less likely to persist in resource-poor settings where these relatively expensive antibiotics are rarely used,” noted Mabey [11].

At the consultation, WHO made it crystal clear that it wants to tightly hold the yaws initiative's leadership and to move as quick as possible into action, following a very pragmatic agenda. This means that no large alliances or partnerships with other public and/or private institutions will be envisaged, at least at the kick-off phase of the new project, both for saving time and skipping organizational complications. Rather, a fair share of autonomy will be granted to national governments on how better to implement the eradication strategy, eventually seeking help on their own. Also, the possible synergy and co-implementation of yaws actions with those tackling other “end of the road” diseases such as trachoma (which is also cured using azithromycin; see www.trachoma.org) and helminthiases (lymphatic filariasis, onchocerciasis, schistosomiasis, soil-transmitted helminthiases), and with ongoing vaccination campaigns, will be similarly evaluated in a second moment.

Besides political commitment, a critical issue relates to the costs of eradication campaign and how the needed funds will be raised. Both at the Morges consultation and in the ensuing official papers, this aspect has not been touched in-depth. Many of those present at the meeting are confident that it would be possible to secure funding, now that neglected tropical diseases have a high profile within the WHO and are thus back in the international health agenda [12]. Azithromycin is available internationally in low-cost generic preparations, and costs related to drug acquisition and administration of azithromycin may be even lower than those of the classic treatment for yaws [15]. But, given the large number of people to be treated, a donation program would be an essential ingredient of the new eradication campaign. It is thus of the outmost importance for WHO to tackle this problem rapidly, since leaving countries alone to implement the new strategy can turn out to be a serious limitation, especially in such a global crisis context.

One cannot help but recalling that the most advanced eradication programme, targeting Guinea worm (dracunculiasis), is basically in the hands of the Carter Center in Atlanta (United States), which recently received $40 million in donations from the Bill & Melinda Gates Foundation to complete its job. In 2011, cases of Guinea worm disease occurred in three remaining endemic nations—South Sudan, Mali, and Ethiopia—and in Chad, where there was an isolated outbreak. Just 1,060 cases were reported in 2011—a 41% decrease from 2010—of which 1,030 in South Sudan (www.cartercenter.org). Keeping this pace, eradication could be achieved by 2015. If the yaws campaign takes off and strengthens up, it could well largely benefit from the looming success of the Guinea worm initiative, possibly being the third human disease to be eradicated ever (and the first bacterial one), and bringing again the WHO at the forefront of future action.

How realistic are the 2017/2020 targets for global yaws eradication? If one weighs carefully the current situation and the challenges noted above, even in light of the new azithromycin option, it is highly improbable that the program could be kept on schedule. Expanding the view to other elimination/eradication campaigns, basically all of them lay behind the target date for completion established at their beginning. However, “target dates” are just that, with their psychological appeal, and their rationale being that of framing a herculean task while trying to concentrate general attention for a range of time, and all programs come with the option for adaptation to new conditions and possibly delay built in, even if this is rarely stated. The eradication approach itself is questionable. “I am wary of announcing a global eradication campaign. A step-by-step, incremental approach seems to me better. This posits a regional/national targeted effort, which aims for the interruption of transmission, which in turn might be certified as a regional eradication,” said Stepan. “Being too ambitious about yaws eradication globally tends to put the main initiative in the hands of outside agencies, rather than national agencies, and can leave the disease effort disconnected from other health services in the country.”

## Conclusion

“Eradication will no doubt continue to keep a place in the arsenal of possible public health interventions but, in my view, eradication campaigns should be exceptional and rare,” wrote Stepan [1]. Even for those not particularly fond of the eradication approach, yaws certainly belongs to the exceptions. Yaws is a “low-hanging fruit,” a weakened, vanquishable enemy, given that those engaging in battle will have carved in stone that they will not retreat until the very last case is chased down and cured. India's attainment makes both a splendid example and a benchmark, especially because it was gained even without using azithromycin. WHO remains with the greatest responsibilities: to set up and implement immediate action and to keep national governments on the right track toward eradication. Should the first, inevitably unsteady, steps of the newborn campaign lead to the right path, it is easy to forecast that general enthusiasm will grow and so will advocacy and support.
